# Population pharmacokinetics and exposure-response analysis of levofloxacin in Chinese pediatric patients with severe refractory *Mycoplasma pneumoniae* pneumonia

**DOI:** 10.1128/aac.01853-25

**Published:** 2026-04-20

**Authors:** Xingru Tao, Yuxue Zhou, Shasha Xu, Yanyan Su, Yu Tang, Shengnan Zhang, Zhao Chang, Feifan Xie, Meng Lv

**Affiliations:** 1 Department of Pharmacy, Children's Hospital Affiliated to Zhengzhou University 12636, Zhengzhou, China; 2 Department of Respiratory Medicine, Children's Hospital Affiliated to Zhengzhou University 12636, Zhengzhou, China; 3 Division of Biopharmaceutics and Pharmacokinetics, Xiangya School of Pharmaceutical Sciences, Central South University 618331, Changsha, China; Providence Portland Medical Center, Portland, Oregon, USA

**Keywords:** population pharmacokinetics, exposure-response analysis, levofloxacin, pediatric patients, severe refractory *Mycoplasma pneumoniae *pneumonia

## Abstract

Levofloxacin is recommended as an optional treatment for pediatric patients with severe refractory *Mycoplasma pneumoniae* pneumonia (SRMPP), but its use remains off-label due to limited pharmacokinetic/pharmacodynamic (PK/PD) data in this population. This study aimed to characterize the PK/PD properties of levofloxacin in pediatric SRMPP patients and define optimal dosing regimens. Scavenged blood samples from patients aged ≤18 years were collected using opportunistic sampling strategy to develop a population pharmacokinetic (PopPK) model of levofloxacin. The Cox proportional hazards model was applied to identify the optimal AUC_ss,0–24h_ cutoff associated with improved efficacy (shorter levofloxacin treatment duration and shorter cough resolution). Monte Carlo simulations were then performed to evaluate target attainment for this efficacy cutoff under guideline regimens (8–10 mg/kg q12 h for ages 1–5 years; 8–10 mg/kg q24 h for ages 5–16 years). A total of 191 pediatric patients (0.16–16 years) with SRMPP receiving intravenous levofloxacin were included in the PK analysis, and 161 patients were included in the exposure-response analysis. A two-compartment PopPK model was developed, identifying body weight as a significant covariate on clearance and central volume of distribution, and serum creatinine as an additional covariate on clearance. Patients with AUC_ss,0–24h_ ≥ 30.74 mg·h/L achieved shorter levofloxacin treatment duration (*P* = 0.019). Guideline-recommended dosing regimens result in overexposure in children <5 years and underexposure in those ≥5 years. Our model-based analysis suggests that 11 mg/kg q24 h or 5.5 mg/kg q12 h may provide more appropriate dosing regimens for children aged 1–16 years for the treatment of SRMPP.

## INTRODUCTION

*Mycoplasma pneumoniae* pneumonia (MPP) is a major cause of community-acquired pneumonia (CAP) in children and young adults worldwide (1, 2). Although generally considered mild and self-limiting, recent evidence indicates a rapid global rise in macrolide-resistant *Mycoplasma pneumoniae* (3, 4), which has led to severe refractory MPP (SRMPP) and serious complications (5). Notably, the prevalence of SRMPP has increased with the re-emergence of MPP following the easing of COVID-19 restrictions (6, 7), posing a significant therapeutic challenge in pediatric patients.

Macrolides are the first-line treatment for children and adolescents owing to their favorable safety profile. Tetracyclines (e.g., doxycycline and minocycline) and fluoroquinolones (e.g., levofloxacin and moxifloxacin) are considered alternatives for macrolide-resistant or refractory cases, such as SRMPP (8). A comprehensive network meta-analysis demonstrated that levofloxacin was the most effective option among tetracyclines and fluoroquinolones for *Mycoplasma pneumoniae* infections (9), and it is now frequently used in China for pediatric patients with SRMPP. Traditionally, fluoroquinolones have been reserved for adolescents and adults due to concerns regarding musculoskeletal toxicity (e.g., cartilage injury) observed in juvenile animal studies (10, 11). However, recent clinical evidence indicates that fluoroquinolones, including levofloxacin, are generally well tolerated in young children, with cartilage-related adverse effects being rare or reversible (12–16).

Levofloxacin is currently used off-label as an intravenous administration in Chinese children with SRMPP, but the optimal dosing regimen has not been established. The recommended dosing regimens by guidelines are extrapolated from a pediatric pharmacokinetics (PK) study of levofloxacin (17), in which dosing was derived from an exposure-matching strategy to achieve a similar area under the concentration-time curve (AUC) to that observed in adults with bacterial infections. Specifically, the guidelines-recommended dosing regimens consist of 8–10 mg/kg every 12 h for children aged 6 months to <5 years, and 8–10 mg/kg every 24 h for those aged ≥5 years (8, 18). However, whether these off-label regimens result in under- or overexposure in Chinese children with SRMPP remains uncertain, as no pharmacokinetic/pharmacodynamic (PK/PD) data are currently available in this population. To date, the pharmacokinetics of oral levofloxacin have been primarily investigated in African pediatric patients with drug-resistant tuberculosis (Table S1) (19–24). Levofloxacin exhibits concentration-dependent antibacterial activity, with the ratio of the 24-h AUC at steady state to the minimum inhibitory concentration (AUC_ss,0–24h_/MIC) strongly associated with clinical efficacy (25–28). Nevertheless, the PK/PD targets of levofloxacin for treating SRMPP in children have yet to be defined.

This study aimed to characterize the population pharmacokinetics and exposure-response relationship of intravenous levofloxacin in Chinese pediatric patients with SRMPP to guide evidence-based dosing in this population.

## MATERIALS AND METHODS

### Study design and ethics

This was a single-center, prospective, open-label PK/PD study of levofloxacin in pediatric patients (≤18 years) diagnosed with or suspected of SRMPP at the Children’s Hospital of Zhengzhou University between April 2023 and April 2024. SRMPP was defined according to the 2023 Chinese guidelines (8). The protocol was approved by the hospital ethics committee (2022-K-L061), and written informed consent was obtained from parents.

### Levofloxacin dosing and concentration analysis

Levofloxacin was administered intravenously at 8–10 mg/kg per dose, once daily in patients ≥5 years and twice daily in those <5 years, not exceeding 750 mg/day, with each dose infused over 0.5–2 h *via* a syringe pump. Blood samples were collected using a scavenged opportunistic approach, centrifuged at 5,000 rpm for 5 min at 4°C, and plasma was stored at −70°C for up to 30 days. Levofloxacin concentrations were determined using a validated HPLC-MS/MS method (29).

Patient data, including age, height, body weight (BW), gender, aspartate aminotransferase (AST), alanine aminotransferase (ALT), total bilirubin (TBIL), direct bilirubin (DBIL), serum albumin (ALB), and serum creatinine (SCr), were collected from medical records. The estimated glomerular filtration rate (eGFR) was calculated by the modified Schwartz equation (30).

### Evaluation of clinical efficacy and safety of levofloxacin

Clinical efficacy of intravenous levofloxacin was evaluated based on (i) clinical response rate, (ii) duration of levofloxacin therapy calculated from the start of its therapy, (iii) time to cough resolution after levofloxacin therapy, and (iv) total hospitalization duration. Clinical response was defined as cure (complete resolution of baseline signs and symptoms) or improvement (marked reduction of infection-related manifestations), whereas treatment failure was defined as lack of response or worsening of the condition. Because all patients received concomitant glucocorticoids, time to defervescence and fever resolution within 48 h were excluded to avoid confounding.

Safety was assessed based on the incidence of adverse effects, including rash, musculoskeletal disorders (arthralgia, arthritis, gait disturbance, tendinopathy, and tendon rupture), and other adverse events.

### Population pharmacokinetic modeling

Population pharmacokinetic (PopPK) analysis of levofloxacin was conducted using Phoenix NLME (version 8.5, Certara, St. Louis, USA) with the first-order conditional estimation-extended least squares method. Interindividual variability of PK parameters was modeled exponentially, and residual variability was assessed using additive, proportional, and combined error models. Covariates were screened by stepwise forward inclusion (*P* < 0.01) and backward elimination (*P* < 0.001) (31). Model performance was evaluated through goodness-of-fit (GOF) plots, nonparametric bootstrap, and visual predictive checks (VPC) to ensure reliability and robustness of the final PopPK model.

### Exposure-response analysis

The AUC_ss,0–24h_ of levofloxacin for all participants at steady state was estimated by the maximum a posteriori Bayesian (MAPB) forecasting method, that is, the individual PK parameters were generated based on the final PopPK model and the observed concentrations, and then the total concentration data for each patient were obtained and AUC_ss,0-24h_ was calculated using non-compartment analysis. Exposure-response analysis was conducted using both graphical and numerical approaches. Separate Cox proportional hazards models were applied to identify the optimal cutoff value of levofloxacin AUC_ss,0–24h_ for levofloxacin treatment duration as the primary outcome and time to cough resolution as the secondary outcome, respectively. Univariable Cox proportional hazards models were employed to identify predictors including patient demographics (e.g., age), clinical laboratory parameters (e.g., ALT), and complications (e.g., pulmonary necrosis) as significant risk factors for clinical outcomes. Statistically significant factors (*P* < 0.05) in univariable analysis were subsequently incorporated into the multivariable Cox proportional hazard model to adjust for confounding and identify independent risk factors. AUC_ss,0–24h_ was classified using a percentile grid spanning the 1st to the 99th percentiles as the candidate cutoff, with values ≥ cutoff defined as the “High AUC” group and values < cutoff as the “Low AUC” group. And then AUC_ss,0–24h_ was modeled as a binary variable within the final Cox model. The cutoff corresponding to the maximum log-likelihood was selected, representing the strongest separation in hazard profiles between groups. Cumulative events of levofloxacin discontinuation were evaluated by inverse probability of treatment weighting (IPTW)-adjusted Kaplan-Meier analysis. All analyses were conducted in R (version 4.0.5) using the survival, survminer, and maxstat packages*.*

### Monte Carlo simulations

Monte Carlo simulations were performed to predict the levofloxacin probability of target attainment (PTA) and risk of toxicity in pediatric patients. Intravenous levofloxacin regimens (8, 9, and 10 mg/kg q12 h for children aged 1–5 years; 8, 9, and 10 mg/kg q24 h for children aged 5–16 years) were evaluated. Specific clinical scenarios based on the characteristics of our modeling population were constructed, and simulations (*n* = 1,000 per scenario) were conducted to generate predicted concentration-time profiles, from which AUC_ss,0-24h_ and probabilities of achieving PK/PD targets were calculated. The AUC_ss,0–24h_ cutoff for improved clinical efficacy was used as the efficacy threshold, while 108 mg·h/L, corresponding to the highest clinically used adult dose of 750 mg every 24 h, was set as the safety threshold. Optimal dosing regimens were defined as those achieving ≥90% PTA for efficacy while maintaining <10% risk of toxicity.

## RESULTS

### Patient demographics for PK/PD analysis

A total of 191 pediatric patients with SRMPP (100 males and 91 females) receiving intravenous levofloxacin were included for PK analysis. Demographics and laboratory variables were summarized in Table 1. The mean age and BW were 7.07 ± 2.77 years and 24.05 ± 9.00 kg, respectively. Patients received a mean daily dose of 11.32 ± 3.06 mg/kg, administered once or twice daily, without dose adjustments during treatment.

**TABLE 1:** Baseline characteristics and dosing information of pediatric patients included in the pharmacokinetic analysis (*n* = 191)^*a*^

Characteristics	Mean ± SD	Median (IQR)
Male/female (n [%])		100 (52.36)/91 (47.64)
Age (years)	7.07 ± 2.77	6.92 (5.25, 8.58)
Body weight (kg)	24.05 ± 9.00	22.50 (18.00, 28.00)
Albumin (g/L)	38.72 ± 4.98	38.80 (35.80, 42.20)
Aspartate aminotransferase (U/L)	34.87 ± 26.14	28.20 (21.10, 38.40)
Alanine aminotransferase (U/L)	36.39 ± 54.24	19.20 (12.40, 34.70)
Direct bilirubin (μmol/L)	1.86 ± 0.68	1.80 (1.40, 2.30)
Total bilirubin (μmol/L)	5.71 ± 2.22	5.30 (4.20, 6.80)
Alkaline phosphatase (U/L)	150.32 ± 55.82	140.70 (115.70, 173.70)
Serum creatinine (μmol/L)	31.77 ± 8.03	30.70 (26.80, 37.00)
Estimated glomerular filtration rate (mL/min/1.73m^2^)	149.61 ± 34.48	144.30 (125.58, 167.43)
Blood urea nitrogen (mmol/L)	3.75 ± 1.08	3.50 (3.10, 4.30)
Levofloxacin daily dose (mg)	259.17 ± 83.13	244.00 (200.00, 290.00)
Levofloxacin daily dose (mg/kg)	11.32 ± 3.06	10.00 (9.83, 10.04)

^*a*^ SD: standard deviation. IQR: interquartile range. The estimated glomerular filtration rate (eGFR) was calculated by the modified Schwartz equation.

For the PD analysis, 165 patients with complete clinical efficacy and safety records were included, with their demographics and laboratory variables presented in Table S2.

### PopPK analysis of levofloxacin

A total of 293 scavenged blood samples were collected for PopPK analysis, with the plasma concentration-time profile shown in Fig. S1. A two-compartment model with first-order elimination best described the data. Interindividual variability for the central (V_1_) and peripheral (V_2_) volumes of distribution and intercompartmental clearance was fixed to zero due to high shrinkage. Residual variability was best captured by a proportional model.

Correlations between every two potential covariates listed in Table 1, with the exception of sex, were calculated by using the Pearson method and elucidated in Fig. S2. Considering the high correlation of BW with age and height (r = 0.84 and 0.88) (Fig. S2), BW was the single covariate that entered into the stepwise screening process to avoid a collinearity effect. In the final model, BW significantly influenced both V_1_ and clearance (CL), with the relationships best described by power models. SCr was also identified as a significant covariate for CL, likewise modeled using a power function. Other tested covariates, including AST, ALT, TBIL, DBIL, ALB, and eGFR, were not significant. Final PK parameters are presented in Table 2.

**TABLE 2:** Population pharmacokinetic estimates of levofloxacin for the final model and bootstrap results^*a*^

Parameter	Final model estimate (RSE [%])	Bootstrap results median (95% CI)
Fixed effects		
θ_CL_ (L/h)	6.85 (4.33%)	6.88 (6.34, 7.48)
θ_BW_CL_	1.11 (12.88%)	1.09 (0.76, 1.29)
θ_SCr_CL_	−0.20 (24.86%)	−0.20 (−0.31,−0.10)
θ_V1_ (L)	25.04 (8.78%)	25.15 (20.98, 29.94)
θ_BW_V1_	1.83 (14.66%)	1.77 (1.24, 2.18)
θ_Q_ (L/h)	1.20 (29.54%)	1.19 (0.64, 2.72)
θ_V2_ (L)	5.28 (18.84%)	5.32 (3.51, 8.38)
Interindividual variability		
CL (ω^2^)	0.013 (21.08%)	0.012 (0.0073, 0.018)
Residual variability		
Proportional error (%)	35.0 (5.90%)	35.0 (31.0, 39.0)

^*a*^ θ_CL_ is the population estimate of clearance (CL), and θ_V1_ is the population estimate of central volume of distribution (V_1_). θ_Q_ is the population estimate of intercompartmental clearance (Q), and θ_V2_ is the population estimate of peripheral volume of distribution (V_2_). θ_BW_CL _and θ_SCr_CL_ represent body weight (BW) and serum creatinine (SCr) related changes on CL, respectively. θ_BW_V1 _indicates body weight-related change on V_1_. RSE, relative standard error. CI, confidence interval. $\begin{array}{rl} & CL=\theta_{CL}\cdot (BW/24.05{)}^{\theta_{BW\_CL}}\cdot (SCr/31.77{)}^{\theta_{SCr\_CL}}\cdot e^{\eta_{CL}}\\ & V_{1}=\theta_{V_{l}}\cdot (BW/24.05{)}^{\theta_{BW\_V1}} \end{array}$.

**Fig 1:**
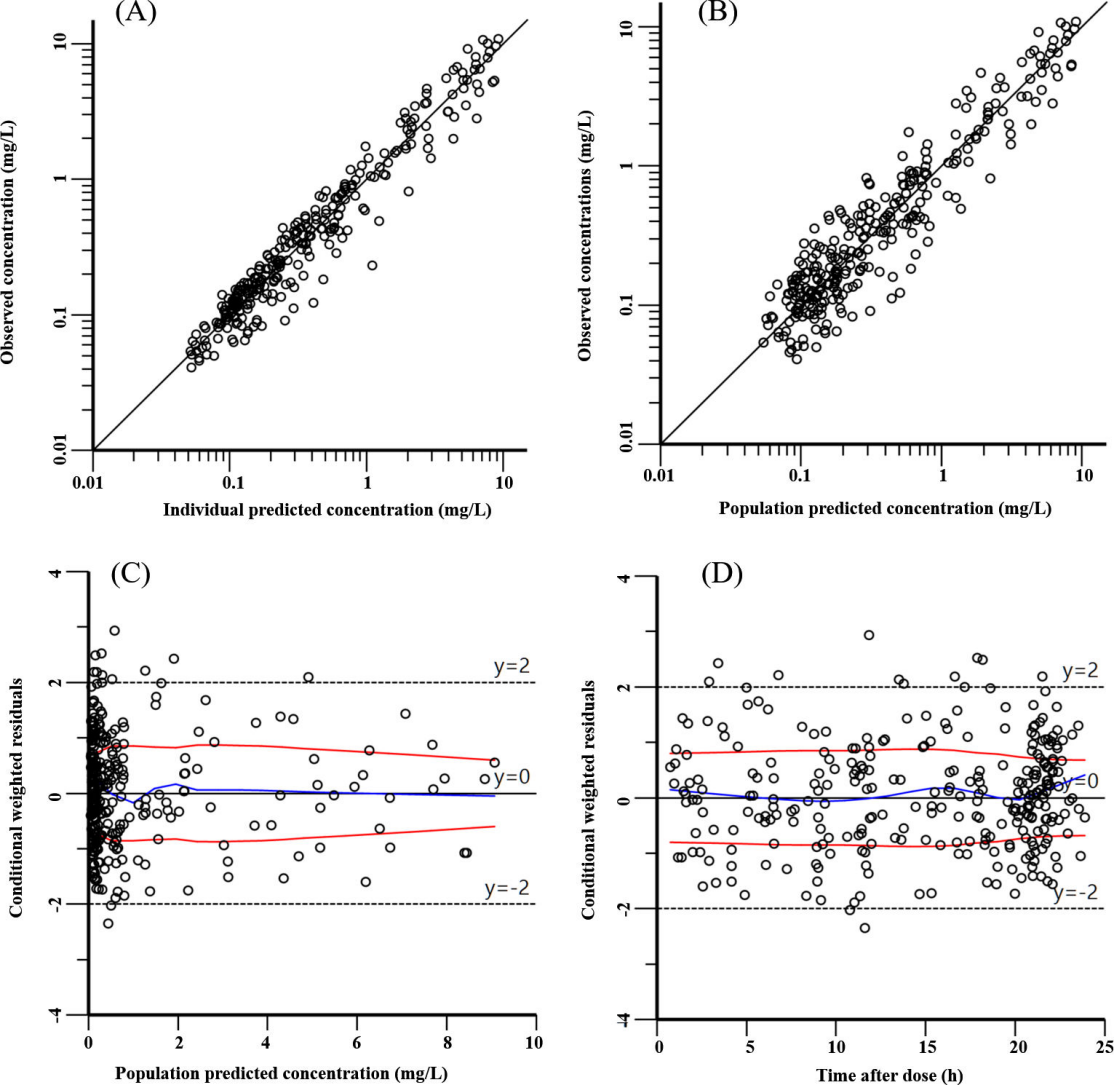
Goodness-of-fit plots of the final levofloxacin PopPK model. (**A**) Individual observed versus predicted concentrations. (**B**) Population observed versus predicted concentrations. (**C**) Conditional weighted residuals versus population predicted concentrations. (**D**) Conditional weighted residuals versus time after dose. In Panels **A and B**, the black solid lines represent the y = x identity lines. In Panels **C and D**, the blue solid lines represent locally weighted scatterplot smoothing (LOESS) curves for the overall conditional weighted residuals, and the red solid lines represent LOESS curves for the absolute values of the overall conditional weighted residuals and their reflection through the x-axis.

**Fig 2:**
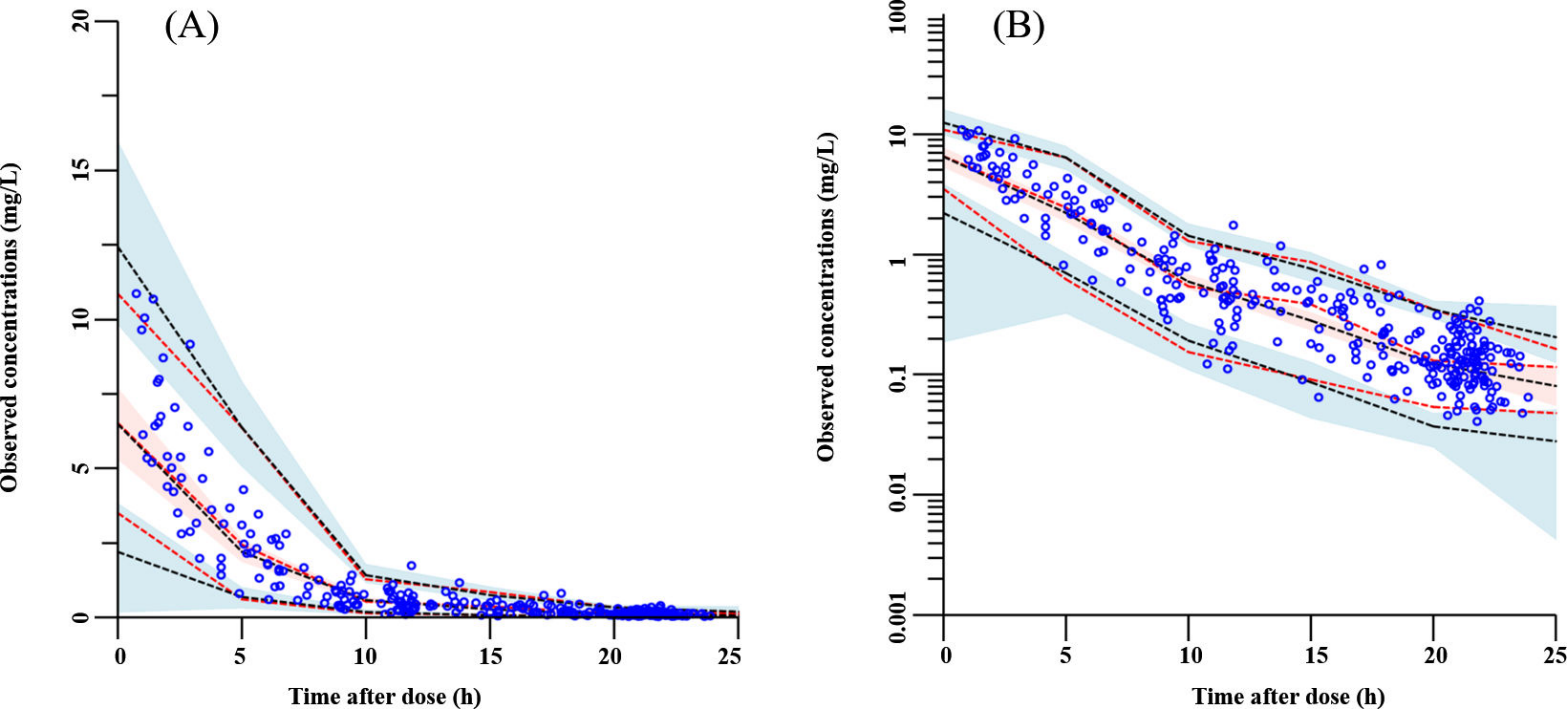
Visual predictive check plots of the final levofloxacin PopPK model. (**A**) Linear plot; (**B**) semi-log plot. Red lines indicate the 5th, 50th, and 95th percentiles of the observed data, while black lines represent the corresponding percentiles of the simulated concentrations. Shaded areas depict the 95% confidence intervals for the simulated percentiles.

The GOF plots (Fig. 1) and VPC plots (Fig. 2) demonstrated good agreement between the model predictions and observed data. Bootstrap results (Table 2) further confirmed the robustness of the final model estimates.

### Exposure-response analysis

Among the 165 patients included in the clinical efficacy and safety analysis, four discontinued levofloxacin due to adverse effects (e.g., arthralgia), which subsequently resolved (Table S2). These patients were excluded from efficacy assessment, and the remaining 161 patients all achieved clinical cure or significant improvement at discharge.

Exploratory visualizations using LOESS-smoothed scatter plots were performed to characterize the relationship between levofloxacin exposure and clinical outcomes. As shown in Fig. S3, a negative trend was observed between AUC_ss,0–24h_ versus levofloxacin treatment duration and time to cough resolution after levofloxacin therapy, which is consistent with the established PK/PD properties of levofloxacin.

**Fig 3:**
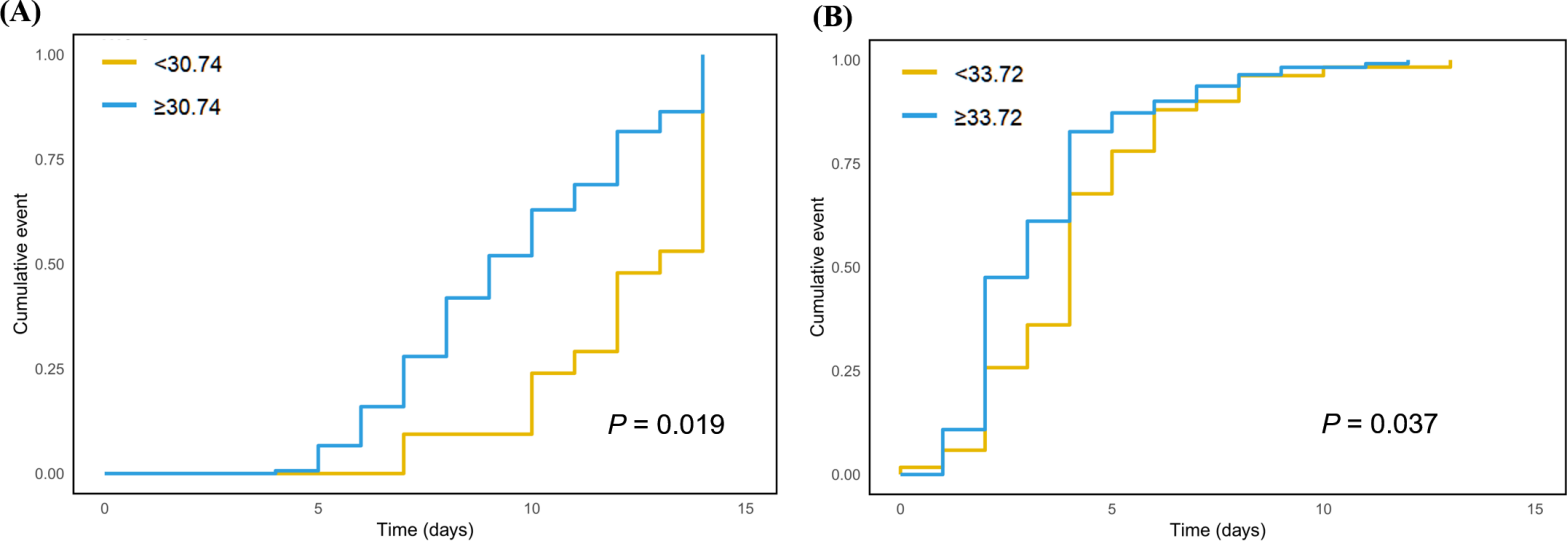
Inverse probability of treatment weighting (IPTW)-adjusted Kaplan-Meier curves of (**A**) cumulative levofloxacin discontinuation events by AUC_ss,0–24h_ ≥ 30.74 versus < 30.74 mg·H/L, and (**B**) cumulative cough resolution events by AUC_ss,0–24h_ ≥ 33.72 versus < 33.72 mg·h/L.

Using the Cox proportional hazards model, pulmonary necrosis was identified as a significant independent factor affecting levofloxacin treatment duration (*P* = 0.02). After adjusting for pulmonary necrosis, an optimal levofloxacin AUC_ss,0–24h_ cutoff of 30.74 mg·h/L was established for shorter levofloxacin treatment duration (HR = 2.03, 95% CI = 1.12–3.68, *P* = 0.019). Kaplan-Meier analysis confirmed significantly shorter levofloxacin therapy in patients with AUC_ss,0–24h_ ≥ 30.74 mg·h/L compared to those below this threshold (Fig. 3A). Similarly, pulmonary embolism was an independent significant factor affecting the time to cough resolution after levofloxacin therapy (*P* = 0.02). After adjusting for pulmonary embolism, an optimal levofloxacin AUC_ss,0–24h_ cutoff of 33.72 mg·h/L was determined for faster cough resolution (HR = 1.43, 95%CI = 1.02–2.00, *P* = 0.037), which was further demonstrated by Kaplan-Meier analysis (Fig. 3B).

Patients with AUC_ss,0–24h_ ≥ 30.74 mg·h/L had a significantly shorter levofloxacin treatment duration (mean = 9.53 days, SD = 2.82 days) compared with those with AUC_ss,0–24h_ < 30.74 mg·h/L (mean = 12.10 days, SD = 2.23 days) (Wilcoxon rank-sum test *P* < 0.01, Fig. S4A). Similarly, patients with AUC_ss,0–24h_ ≥ 33.72 mg·h/L had a significantly shorter time to cough resolution (mean = 3.34 days, SD = 2.14 days) than those with AUC_ss,0–24h_ < 33.72 mg·h/L (mean = 4.20 days, SD = 2.37 days) (Wilcoxon rank-sum test, *P* < 0.01, Fig. S4B).

### Monte Carlo simulations

As our study population primarily consisted of children aged 1–16 years (with only one infant under 1 year old, who was excluded to maintain homogeneity), four age groups were considered: 1–3, 3–5, 5–10, and 10–16 years. Within each age group, the 25th, 50th, and 75th percentiles for both BW and SCr were calculated and paired to create representative scenarios (e.g., 25th BW with 25th SCr, 50th with 50th, and 75th with 75th) for each age group, yielding a total of 12 distinct clinical scenarios (Table 3). In children aged 1–5 years, all simulated regimens (8–10 mg/kg q12 h) consistently achieved 100% attainment of the target AUC_ss,0–24h_ (30.74 mg·h/L), regardless of differences in BW or SCr (Table 3). By contrast, in children aged 5–16 years, target attainment was highly regimen- and covariate-dependent. Dosing regimens of 8–9 mg/kg q24h achieved only 14.6%–62.9% attainment, whereas 10 mg/kg q24 h approached relatively acceptable levels (80%–90%). For both age groups, 11 mg/kg q24 h or 5.5 mg/kg q12 h consistently achieved ≥90% target attainment (Table S3). Notably, across all dosing strategies and patient groups, no simulated regimen exceeded the safety threshold of 108 mg·h/L, suggesting a low risk of toxicity.

**TABLE 3:** Simulated scenarios stratified by body weight and serum creatinine, based on study population characteristics, with probability of target attainment (PTA) for an AUC_ss,0–24h_ target of 30.74 mg·h/L under guideline-recommended dosing regimens

Simulated scenarios	Age (years)	Body weight (kg)	Serum creatinine (μmol/L)	PTA (%)		
				8 mg/kg q12 h	9 mg/kg q12 h	10 mg/kg q12 h
1	1–3	9	19	100	100	100
2		11	21	100	100	100
3		13	28	100	100	100
4	3–5	15	24	100	100	100
5		17	28	100	100	100
6		19	30	100	100	100

Simulated scenarios	Age (years)	Body weight (kg)	Serum creatinine (μmol/L)	PTA (%)		
				8 mg/kg q24 h	9 mg/kg q24 h	10 mg/kg q24 h
7	5–10	21	27	25.7	50.2	84.4
8		24	31	14.6	53.3	83.9
9		27	37	19.1	62.9	88.8
10	10–16	33	34	23.2	48.3	79.6
11		39	41	14.6	54.6	84.9
12		45	45	19.3	55.5	86.4

## DISCUSSION

Levofloxacin is widely used to treat various infectious diseases and is recommended off-label as an alternative therapy for pediatric patients with SRMPP (8, 32). Defining the pharmacokinetics and exposure-response relationship is critical to optimize dosing, maximize efficacy, and minimize toxicity in this vulnerable population.

In this study, we developed, for the first time, a PopPK model of levofloxacin in 191 pediatric patients with SRMPP (aged 0.16–16.0 years) using a scavenged opportunistic sampling approach. A two-compartment model with first-order elimination best described the concentration-time data in our study. This finding is consistent with oral levofloxacin studies in pediatric patients with drug-resistant tuberculosis (21–23). However, depending on sample size and sampling design, some studies have identified a one-compartment model as more suitable (19, 20, 24). In our cohort of Chinese pediatric patients (mean age, 7.07 years; mean body weight, 24.05 kg), the typical weight-normalized CL of levofloxacin following intravenous administration was 0.28 L/kg/h. This value is consistent with reported oral CL estimates in African children, which range from 0.16 to 0.39 L/kg/h across a mean age of 2–9 years (Table S1). Because levofloxacin is rapidly and almost completely absorbed, with an absolute bioavailability close to 100%, intravenous and oral CL values can be directly compared.

BW, the primary descriptor of body size in children, was identified as a significant covariate for both CL and central volume of distribution (V_1_) of levofloxacin, consistent with previous reports (Table S1). In contrast, age was not a significant predictor of CL in our study, although some studies have reported age-dependent changes in levofloxacin clearance (21–24). The inclusion of maturation of CL with age did not further improve the data fit in our study, which may be attributed to the small sample size under 2 years (7 patients with 11 concentrations) and the interindividual variability could be well explained by the included covariates. We also found that SCr, a marker of renal function, significantly influenced CL, whereas eGFR did not retain significance in the final model. This finding confirms that renal function is an important determinant of levofloxacin elimination in children, which is consistent with observations in adults and physiologically expected, given that levofloxacin is almost entirely eliminated unchanged *via* the kidneys (33). During covariate screening, both SCr and eGFR individually led to a significant drop in the objective function value (OFV) when added to the base model. However, the inclusion of SCr resulted in a slightly greater reduction in OFV compared to eGFR (−216.15 versus −215.89). The relatively narrow range of eGFR in our study population probably accounted for its weaker association with CL. Taking into account both statistical performance and clinical practicality, SCr was ultimately selected as the covariate for CL in the final model.

The duration of levofloxacin therapy was chosen as the primary efficacy outcome, as it reflects the time required for complete resolution of baseline signs and symptoms. Time to cough resolution was selected as the secondary outcome, serving as a sensitive indicator of improvement in infection-related manifestations. Total hospitalization duration was not included in the Cox proportional hazards model, since hospitalization time before initiation of levofloxacin varied among patients and thus was not a reliable measure of levofloxacin efficacy. Exposure-response analysis showed that the AUC_ss,0–24h_ cutoffs for shorter levofloxacin therapy and faster cough resolution were similar (30.74 versus 33.72 mg·h/L), and 30.74 mg·h/L was selected as the AUC target for model-based simulations to identify optimal dosing regimens. Our simulations showed that while the guideline-recommended regimens of 8−10 mg/kg q12 h for children aged 1 to < 5 years achieved the target AUC, it appeared excessive, as further analysis showed that 5.5 mg/kg q12 h or 11 mg/kg q24 h were sufficient to ensure ≥90% target attainment. In contrast, the recommended 8−10 mg/kg q24 h for children aged ≥5 years was inadequate, whereas 11 mg/kg q24 h or 5.5 mg/kg q12 h consistently achieved ≥90% target attainment. Overall, 11 mg/kg q24 h or 5.5 mg/kg q12 h were identified as the optimal regimens for children aged 1−16 years, suggesting that the current age-based cutoff of 5 years for switching between q12 h and q24 h dosing is not justified.

We acknowledge some limitations in this study. First, as a single-center study focusing on pediatric patients with SRMPP, although we enrolled 191 children aged 0.16–16 years, only 7 patients were under 2 years old. Consequently, our recommended levofloxacin dosing regimens should be applied with caution in children younger than 2 years. Second, the use of levofloxacin therapy duration as the primary efficacy outcome in an open-label design, which may be influenced by clinicians’ subjective judgment. Nevertheless, the optimal AUC_ss,0–24h_ cutoff (30.74 mg·h/L) derived from the primary outcome was consistent with that from the more objective, patient-centered secondary outcome of time to cough resolution (33.72 mg·h/L), which confirms the robustness of our AUC_ss,0–24h_ threshold. Moreover, all included patients in the present study received concomitant glucocorticoids as part of standard clinical care. Extrapolation of these findings to patients not receiving glucocorticoids should be performed with caution, as the exposure-response relationship may differ. Third, while no musculoskeletal toxicity was observed in the studied children, our sample size is insufficient to fully characterize the safety profile of levofloxacin in pediatrics. Future large-scale clinical trials are needed to further confirm its safety in this population.

### Conclusion

In conclusion, we developed a PopPK model of levofloxacin in Chinese pediatric patients with SRMPP (aged 0.16−16 years) and identified BW and SCr as significant covariates on clearance. Exposure-response analysis using a Cox proportional hazards model showed that an AUC_ss,0–24h_ ≥ 30.74 mg·h/L was associated with shorter levofloxacin treatment duration. Model-based simulations indicated that 11 mg/kg q24 h or 5.5 mg/kg q12 h are the optimal regimens for children aged 1−16 years.

## Data Availability

The data that support the findings of this study are available on request from the corresponding author on reasonable request.

## SUPPLEMENTAL MATERIAL

Supplemental material aac.01853-25-s0001.docx Tables S1 to S3; Fig. S1 to S4.

ASM does not own the copyrights to Supplemental Material that may be linked to, or accessed through, an article. The authors have granted ASM a non-exclusive, world-wide license to publish the Supplemental Material files. Please contact the corresponding author directly for reuse.
