# Supplementary material for: Population pharmacokinetics and exposure-response analysis of levofloxacin in Chinese pediatric patients with severe refractory Mycoplasma pneumoniae pneumonia
Source: Antimicrob Agents Chemother. 2026 Apr 20;70(6):e01853-25. doi: 10.1128/aac.01853-25 (PMC13231918; doi:10.1128/aac.01853-25)
Supplement: Supplemental material — Tables S1 to S3; Fig. S1 to S4. [file aac.01853-25-s0001.docx]

**Supp****lementary data**

Table S1. Basic study characteristics and pharmacokinetic parameters from reported pediatric levofloxacin pharmacokinetic studies.

| Study | Country | Patient number | Age  (years) ^a^ | Body weight  (kg) ^a^ | Route | Dosing regimen | Model structure | CL/F (L/kg/h) | V/F (L/kg) | Significant covariates |
| --- | --- | --- | --- | --- | --- | --- | --- | --- | --- | --- |
| Savic RM [19] | South Africa | 23 | 3.14  (0.25-8) | / | oral | 15 mg/kg qd | One-compartment | 0.39 | 1.81 | Body weight |
| Mase SR [20] | Federated States of Micronesia and Republic of the Marshall Islands | 50 | 9.3 ± 4.2  (0.5-15) | 29.2 ± 13.1 | oral | 5-20 mg/kg qd | One-compartment | 0.16 | 1.26 | Body weight |
| Denti P [21] | South Africa | 109 | 2.1  (0.3-8.7) | 12  (6-22) | oral | 10-20 mg/kg qd | Two-compartment | 0.39 | 1.6 | Body weight, age, HIV |
| Garcia-Prats AJ [22] | South Africa | 133 | 2.1  (1.2, 3.7) | 12.4  (9.3, 14.7) | oral | 10-21 mg/kg qd | Two-compartment | 0.23 | 1.00 | Body weight, age, HIV |
| van der Laan LE [23] | South Africa | 25 | 2.56  (0.16-6.01) | 12.2  (10.7, 15.0)  4.02-20.3 | oral | 15-20 mg/kg qd | Two-compartment | 0.34 | 1.61 | Body weight, age |
| White YN [24] | Pakistan, South Africa | 242 | 2.8 (0.2-16.3) | 12.9 (4.0-48.5) | oral | 15-25 mg/kg qd | One-compartment | 0.24 | 1.31 | Body weight, age |

^a^ Values are expressed as median, (range) or mean ± standard deviation.

Table S2. Baseline characteristics and clinical response of pediatric patients included in the exposure-response analysis (n=161).

| Characteristics | Median (IQR) or n (%) |  |
| --- | --- | --- |
| Demographics and clinical laboratory tests | |  |
| Male/female | 82 (50.93)/ 79(49.07) |  |
| Age (years) | 6.83 (5.17, 8.58) |  |
| Body weight (kg) | 22.00 (18.00, 26.80) |  |
| Albumin (g/L) | 39.30 (36.50, 42.60) |  |
| Aspartate aminotransferase (U/L) | 27.70 (21.10, 35.00) |  |
| Alanine aminotransferase (U/L) | 18.90 (12.40, 31.00) |  |
| Direct bilirubin (μmol/L) | 1.80 (1.40, 2.30) |  |
| Total bilirubin (μmol/L) | 5.40 (4.20, 6.80) |  |
| Alkaline phosphatase (U/L) | 140.70 (115.10, 172.40) |  |
| Serum creatinine (μmol/L) | 30.70 (26.90, 36.90) |  |
| Estimated glomerular filtration rate (mL/min/1.73m^2^) | 142.97 (125.69 165.97) |  |
| Blood urea nitrogen (mmol/L) | 3.50 (3.00, 4.40) |  |
| White blood cell (×10^9/L) | 9.31 (6.63, 12.02) |  |
| C-reactive protein (mg/L) | 14.89 (5.32, 37.46) |  |
| Procalcitonin (ng/mL) | 0.12 (0.07, 0.26) |  |
| Drug treatment |  |  |
| Duration of macrolide exposure before levofloxacin treatment (days) | 6.00 (4.00, 7.00) |  |
| Levofloxacin daily dose (mg) | 240.00 (200.00, 280.00) |  |
| Levofloxacin daily dose (mg/kg) | 10.00 (9.82, 10.04) |  |
| Levofloxacin AUC_ss,0-24h_ | 35.59 (33.11, 41.54) |  |
| Complications |  |  |
| Pleural effusion | 41 (25.31) |  |
| Pulmonary embolism | 10 (6.17) |  |
| Pulmonary necrosis | 25 (15.43) |  |
| Clinical efficacy |  |  |
| Clinical cure / improvement | 161 (100) |  |
| Time to cough resolution after levofloxacin therapy (days) | 3 (2, 4) |  |
| Duration of levofloxacin therapy (days) | | 10 (7, 12) |
| Total hospitalization duration (days) | 12 (9, 15) |  |
| Adverse effects |  |  |
| Arthralgia | 1 |  |
| Lower limb pain | 2 |  |
| Rash | 1 |  |

IQR: interquartile range. The estimated glomerular filtration rate (eGFR) was calculated by the modified Schwartz equation.

Table S3. Simulated scenarios stratified by body weight and serum creatinine, based on study population characteristics, with probability of target attainment (PTA) for an AUC_ss,0–24h_ target of 30.74 mg·h/L under other dosing regimens.

| Simulated scenarios | Age (years) | Body weight (kg) | Serum creatinine (μmol/L) | PTA (%) | | |
| --- | --- | --- | --- | --- | --- | --- |
|  |  |  |  | 9 mg/kg q24h | 10 mg/kg q24h | 11 mg/kg q24h |
| 1 | 1-3 | 9 | 19 | 60.9 | 88.8 | 98.2 |
| 2 |  | 11 | 21 | 59.3 | 88.4 | 97.4 |
| 3 |  | 13 | 28 | 70.2 | 92.4 | 99.2 |
| 4 | 3-5 | 15 | 24 | 54.3 | 86.3 | 97.6 |
| 5 |  | 17 | 28 | 60.8 | 87.9 | 97.8 |
| 6 |  | 19 | 30 | 64.3 | 90.2 | 98.3 |
| 7 | 5-10 | 21 | 27 | 50.2 | 84.4 | 96.3 |
| 8 |  | 24 | 31 | 53.3 | 83.9 | 97.3 |
| 9 |  | 27 | 37 | 62.9 | 88.8 | 98.0 |
| 10 | 10-16 | 33 | 34 | 48.3 | 79.6 | 95.8 |
| 11 |  | 39 | 41 | 54.6 | 84.9 | 97.7 |
| 12 |  | 45 | 45 | 55.5 | 86.4 | 97.0 |

Dosing regimens of 9 mg/kg q24h and 4.5 mg/kg q12h, 10 mg/kg q24h and 5 mg/kg q12h, and 11 mg/kg q24h and 5.5 mg/kg q12h demonstrated equivalent PTA.


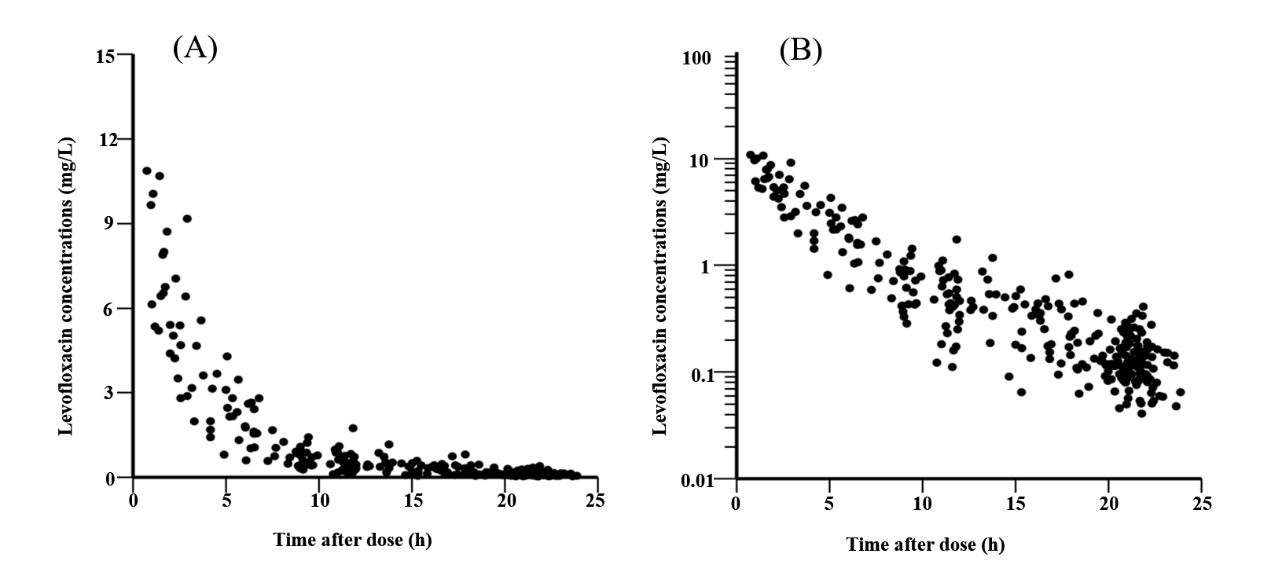
Figure S1. Observed levofloxacin concentrations versus time after the last dose. (A) linear scale; (B) semi-logarithmic scale.


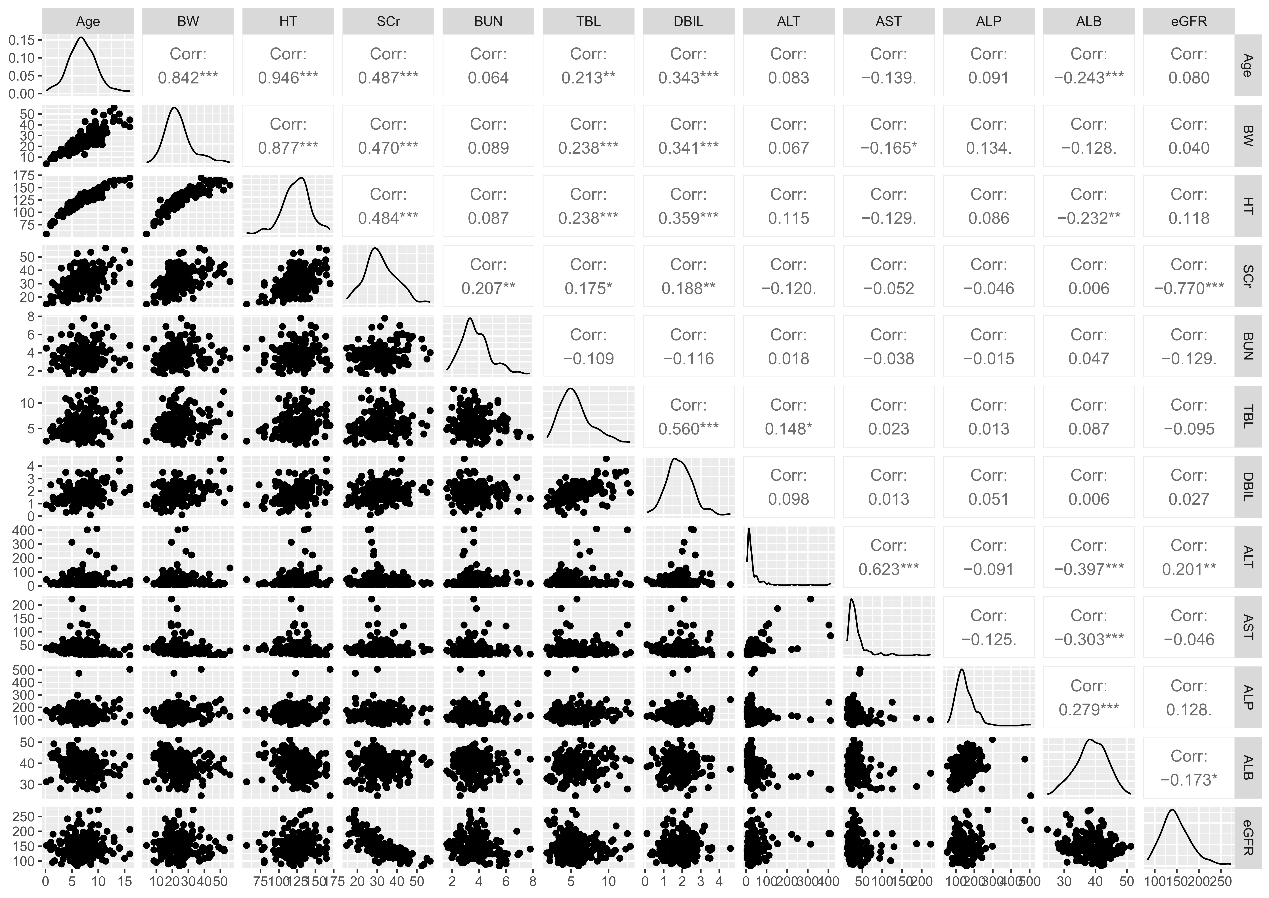


Figure S2. The correlation plot between covariates. BW, body weight; HT, height; SCr, serum creatinine; BUN, blood urea nitrogen; TBL, total bilirubin; DBIL, direct bilirubin; ALT, alanine aminotransferase; AST, aspartate aminotransferase; ALP, alkaline phosphatase; ALB, albumin; eGFR, estimated glomerular filtration rate.


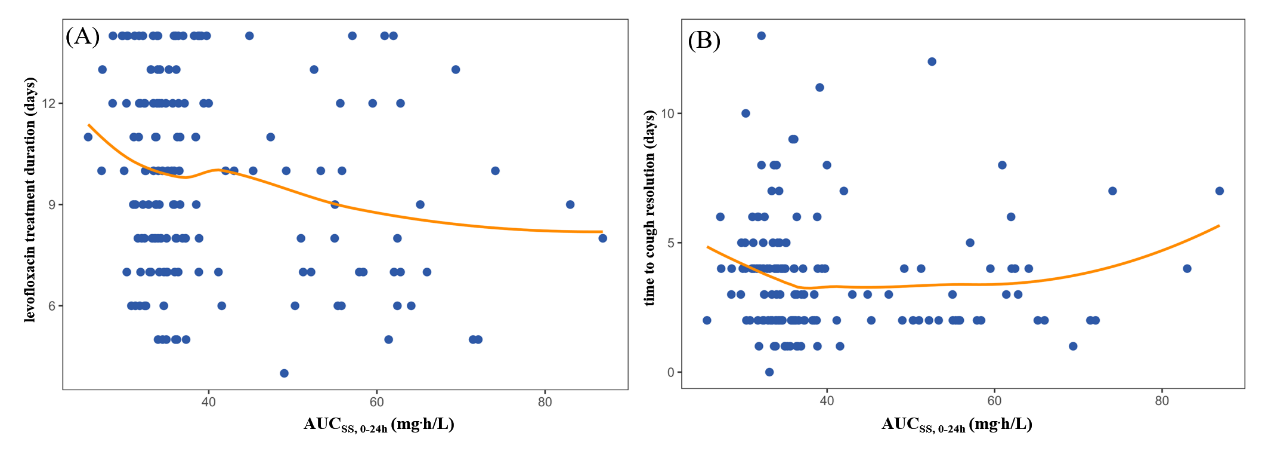


Figure S3. Scatter plots of individual AUC_ss,0-24h_ versus levofloxacin treatment duration (A) and time to cough resolution after levofloxacin therapy, with LOESS smoothing curve (orange line).


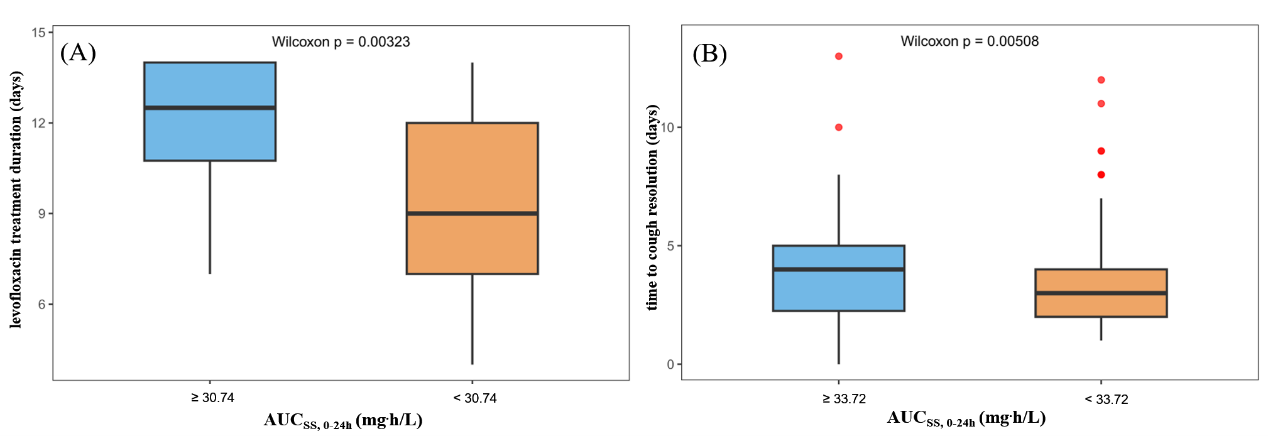


Figure S4. Association between AUC_ss,0-24h_ of levofloxacin and clinical outcomes stratified by cutoff. (A) AUC_ss,0-24h_ of levofloxacin versus levofloxacin treatment duration; (B) AUC_ss,0-24h_ of levofloxacin versus time to cough resolution.
